# Mentoring Support Quality in Group and Individual Mentoring Approaches during Nursing Clinical Training: A Quasi-Experimental Study

**DOI:** 10.3390/nursrep14020065

**Published:** 2024-04-02

**Authors:** Ivana Gusar, Andrea Tokić, Robert Lovrić

**Affiliations:** 1 Department of Health Studies, University of Zadar, 23000 Zadar, Croatia; 2 Department of Psychology, University of Zadar, 23000 Zadar, Croatia; apupic@unizd.hr; 3 Nursing Institute “Prof. Radivoje Radić”, Faculty of Dental Medicine and Health Osijek, Josip Juraj Strossmayer University of Osijek, 31000 Osijek, Croatia; rlovric@fdmz.hr

**Keywords:** quality, mentoring support, nursing, clinical training, mentoring approaches

## Abstract

Clinical training is an essential element in nursing education, the outcomes of which are directly related to the quality of mentoring support. This quasi-experimental study aimed to examine whether the group or individual form of the mentoring approach used and the order of application of the mentoring approach contribute to the quality of mentoring support provided to students. The study comprised two measurement points with 130 nursing students, divided into two groups with different orders of application of the mentoring approach. The validated Mentoring Support Quality Evaluation Questionnaire (MSEQ) was used. Students in both groups rated the quality of mentoring support as higher following an individual mentoring approach. A significant interaction was found between the mentoring approach used and the order in which the mentoring approaches were applied (*p* = 0.002). The individual mentoring approach contributed significantly to a higher quality of mentoring support after the second round of clinical training (*p* = 0.021), while after the first round, the difference between the group and individual approaches was not as clear. The results suggest that not only the form of the mentoring approach but also the sequence of changes in the mentoring approach should be planned when implementing clinical training.

## 1. Introduction

Clinical training is an essential element in nursing education [1] and gives students the chance and responsibility to develop and achieve the competencies required for nursing practice [2]. In accordance with the European directive 2005/36/EC, clinical training is part of nurse training, which implies students’ direct nursing care experiences with patients, or clinical simulation of such experiences, offering the student the opportunity to integrate, apply, and refine specific skills [3]. During clinical training, students are mentored by a mentor who is a qualified registered nurse responsible for providing effective instruction and assessment of undergraduate nursing students in the hospital [2]. Many different mentoring approaches [4,5,6,7,8,9,10] are used worldwide for the clinical education of nursing students, and there are still no clear conclusions and suggestions for the selection of a specific mentoring model as the most appropriate [8], as all mentoring approaches have advantages and disadvantages.

Among the many types of mentoring, group and individual mentoring are most commonly used worldwide [11,12]. The group mentoring approach, in which one mentor assists several students, is effective and practical [6], promotes students’ adaptation to teamwork, and facilitates peer helping [13]. On the other hand, this type of mentoring can lead to an overload for the mentor and a lack of time for the students, which limits the possibility of adapting the learning content to the individual needs of the students [14,15]. The aforementioned deficiencies may negatively affect students’ acquisition of cognitive, psychomotor, and social content and their own professional identification [2]. As a result, students face the problem of not being able to apply previously acquired theoretical knowledge in clinical practice [14,15] and student satisfaction with mentoring is low [2,15]. Relevant literature warns that such situations have a negative impact on students’ motivation to work and on the quality of task performance [16], which in turn can have a negative impact on patient safety and the ultimate quality of healthcare [17]. The use of an individual mentoring approach implies the “one-to-one” relationship, where one mentor supervises one student during clinical training. The individual mentoring approach is mainly related to the effective acquisition of specific knowledge and competencies in the practice of nursing skills by the students [18]. The results of a qualitative longitudinal study conducted in Sweden clearly show that students should not be considered as a group, but as individuals with different prerequisites and learning needs [19]. Furthermore, the individual mentoring approach eliminates some of the main shortcomings and barriers to effective mentoring that exist in the group mentoring approach, such as the overload of the mentor, the lack of time for the students, and the inability to adapt the mentoring programme to the student’s needs. In addition, the individual mentoring approach provides better opportunities for the development of a quality relationship between the mentor and the student, for continuity of care for the student, and most importantly, for the provision of timely feedback, while the student has the opportunity to see the mentor as a role model for their professional development [2]. This form of mentoring approach generally contributes positively to student satisfaction with the mentoring approach, which can also have a positive impact on student motivation and ultimately on the quality of task performance in healthcare [2,11]. In addition, the individual mentoring approach promotes a stronger personal identification with the profession [2,11]. According to the European Union (EU) Directive, nursing students in Croatia, as in other EU Member States, are required to spend at least 4600 h in actual patient situations and practice settings during their three-year undergraduate study, interacting and collaborating with a clinical mentor [20,21], which emphasises the importance of the quality of mentoring processes and support. The quality of mentoring support during clinical training is considered an important factor in the effective achievement of learning outcomes and, more generally, in the academic success of students during the study programme [16]. Furthermore, according to relevant literature, the quality of mentoring support is reflected in students’ continued commitment to their own profession [22], which is particularly important given the global shortage of healthcare professionals, especially nurses [23]. Although Croatia has adopted the EU directive, the system of mentoring in nursing in the Republic of Croatia has not changed significantly compared to the past [2], i.e., the group mentoring approach dominates nursing education in Croatia. 

Although there is a lot of research on clinical training, there is a lack of experimental studies and comparisons of the quality of mentoring support during clinical training in different (group and individual) mentoring approaches. All mentoring approaches have advantages and disadvantages, and the results of previous research are not consistent in terms of concluding which approach is more efficient. To address this global gap and to corroborate the findings of previous studies, the research question of this study was to investigate the contribution of group and individual mentoring approaches to the quality of mentoring support during clinical training, as well as whether the order of application of mentoring contributes to the quality level of mentoring support during clinical training. 

## 2. Materials and Methods

### 2.1. Sampling

Students of each generation (1st, 2nd, and 3rd year of study) were divided into two groups (Group 1 and Group 2). These groups were matched according to their previous study satisfaction (F (1.105) = 0.003; *p* = 0.592) and the average grades achieved (F (1.114) = 0.00; *p* = 0.703), as these two variables can be seen as potential influencing factors for our dependent variable. 

### 2.2. Study Design

The study design was quasi-experimental and involved measuring the quality level of mentoring support (dependent variable) during the mandatory clinical training courses that are regularly conducted during all three years of undergraduate nursing study. All students who participated in the study were exposed to both group and individual mentoring (1st independent variable), while the second independent variable was the order in which they were exposed to the different types of mentoring. The study consisted of two phases. 

**Figure 1:**
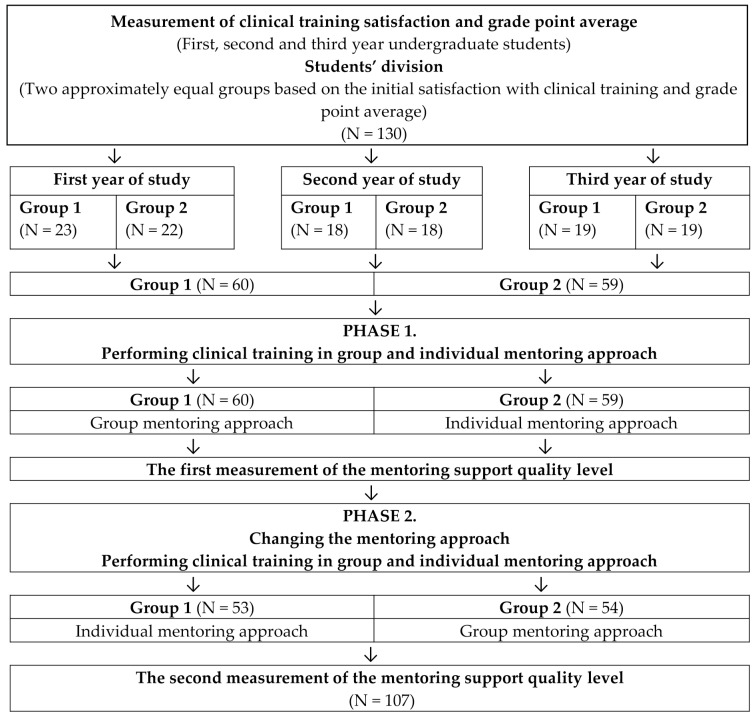
Study design.

In the first phase of the study, all students in Group 1 (n = 60) completed 50 h of clinical training (first round) under a group mentoring approach and students in Group 2 (n = 59) completed 50 h of clinical training (first round) under an individual mentoring approach. The first measurement of the estimated quality level of mentoring support was taken immediately after the second phase. After the first phase, students had a two-month break to neutralise the effects of the previous mentoring approach on the next one, which was applied in the second phase (Figure 1). 

In the second phase of the study, students in Group 1 (n = 53) and Group 2 (n = 54) switched mentoring approaches. Students in Group 1 completed 50 h of clinical training (second round) through an individual mentoring approach, while students in Group 2 were mentored through a group mentoring approach (second round). The second measurement of the estimated quality level of mentoring support was conducted immediately after the second phase (Figure 1).

### 2.3. Participants

The total number of respondents was determined based on the number of students currently enrolled in the undergraduate nursing study. The eligibility criteria for participation in the study were the following: Full-time students in their first, second, or third year of undergraduate nursing studies at a higher education nursing institution in Zadar, Croatia (n = 130); voluntary consent to participate in the study; positively assessed prerequisites for participation in clinical training course 1 for first-year students, clinical training course 2 for second-year students, or clinical training course 3 for third-year students; and completion of at least 50 h of clinical exercises in a hospital department during the first and second phases of the clinical training course.

After the sampling, the number of participants was reduced by 11 students. Four students dropped out, two students declined further participation in the study, and five students did not fulfill the requirements for completion of the clinical training course.

After the first phase of the study, seven students from Group 1 and five students from Group 2 had not completed all 50 h of clinical training and did not continue to participate in the study because they did not fulfill the criteria.

At the end of the study, a total of 107 students (Group 1 = 53; Group 2 = 54) participated and completed both the first and second rounds of clinical training (Figure 1).

### 2.4. Interventions

During the first round of the clinical training course (first research phase), Group 1 students (n = 60) were divided into 12 subgroups and performed 50 h of clinical exercises in a hospital department in a group mentoring approach, where a group of five students was supervised by one mentor. Group 2 students (n = 59) completed clinical training in a hospital department using an individual mentoring approach—one mentor supervised one student at a time (50 h per student).

In the second round of clinical training (the second research phase), Group 1 students (n = 53), who had completed at least 50 h of clinical training in the group mentoring approach in the first round, now performed clinical training in a hospital department with an individual mentoring approach under the supervision of the same mentor as before. One mentor supervised one student at a time. On the other hand, the students in Group 2 (n = 54), who had completed at least 50 h of clinical training under the individual mentoring approach in the first round, were now divided into 12 subgroups and completed 50 h of clinical exercises in a hospital department under a group mentoring approach, where a group of four to five students was supervised by the same mentor as before under the individual mentoring approach.

A total of 12 mentors took part in supervising the students during their clinical training, eleven female (91.7%) mentors and one male (8.3%) mentor. All mentors have a master’s degree and, during their formal education, attended a course that includes education on mentoring students. They received detailed oral instructions about the content, purpose, and duration of the clinical training from the researcher before the research. The average age of the mentor was 44.83 years (SD = 7.04), the average professional experience was 23.08 years (SD = 6.90), and the average mentoring experience was 6.83 years (SD = 3.92).

### 2.5. Data Collection Procedures

A questionnaire to initially assess students’ study satisfaction was administered to all first-, second-, and third-year clinical training students before the start of the courses, while data on students’ grade point averages were collected from the Information System of Higher Education Institutions (ISVU). Questionnaires for examining the quality of mentoring support in the first and second phases of the study (after the first and second rounds of clinical training) were administered to students who had completed the entire 50 h of clinical training immediately after clinical training had been delivered through a one-to-one or group mentoring approach in the clinical department lecture theatre.

### 2.6. Instrument

The research instrument comprised questions on the general characteristics of the respondents and the standardised questionnaire for assessing the quality of mentoring support (MSEQ) by Vizek Vidović et al. [24]. The original version of the MSEQ questionnaire consists of a total of 39 statements, of which 25 refer to mentoring support and 14 to the teachers of theoretical classes. After the initial content analysis and validation of the instrument, and after taking into account the aim of this research, 14 items related to theory teaching were removed from the final version of the questionnaire used. The factor analysis of the 25-item questionnaire confirmed a unidimensional structure with an average inter-correlation between items of 0.73 and high reliability (Cronbach’s α = 0.98) [2]. Respondents rated the quality of mentoring support on a 5-point Likert scale (from 1 = strongly disagree to 5 = completely agree). The final result is the sum of all responses, with a higher score indicating a higher quality level of mentoring support received.

### 2.7. Ethical Considerations and Procedures

This study complied with the ethical principles for research involving human subjects as laid down in the Declaration of Helsinki. Approval was obtained from the Ethics Committee of the Faculty of Medicine, University of Osijek (IRB approval number: 2158-61-07-20-168) before the start of the study. Before the start of the study, all respondent mentors and students were informed about the aim of the study and its details. They were informed in writing and verbally about voluntary participation, data protection, and confidentiality and could withdraw at any time without consequences. A written informed consent form was obtained from each participant and all participants were informed that they could contact the researchers if they had any further questions. The researchers were not directly or indirectly involved in the students’ clinical training.

### 2.8. Data Analysis

Descriptive statistics for nominal variables are presented in the form of proportions and percentages, and numerical data are presented with the arithmetic mean and standard deviation. The normality of the distribution of the numerical variables was tested with the Shapiro–Wilk test. A one-way analysis of variance (ANOVA) was used to test whether the two groups of students formed were equal in average grades and study satisfaction. A two-way analysis of variance (ANOVA) was used to analyse the difference in the quality of mentoring support between the studied groups of students who undertook clinical training in different forms of mentoring approaches (individual and group mentoring), and to assess the effect of the order of application of the different types of mentoring approaches on satisfaction with the quality of mentoring support.

The significance level α = 0.05 was used as a criterion for the statistical significance of the results obtained. STATISTICA software version 14.0.0.15 (TIBCO Software Inc., Palo Alto, CA, USA 2018) was used to analyse the data.

## 3. Results

### 3.1. Sociodemographic Characteristics of the Respondents

Out of the total of 130 participants in this study, 11 (8%) were men and 119 (92.2%) were women. The average participant age (mean) was 22.2 years old (SD = 5.21). There were 51 (39%) first-year students, 37 (28%) second-year students, and 42 (33%) third-year students. 

### 3.2. Quality Levels of Mentoring Support in Group and Individual Mentoring Approaches

The average levels of the quality of mentoring support and their variables in the first and second rounds of clinical training are shown in Table 1.

### 3.3. Differences in the Quality Level of Mentoring Support in the Group and Individual Mentoring Approaches

The results of the ANOVA analysis point to a significant interdependence of the form of the mentoring approach and the order of application of the mentoring approach on the level of quality of mentoring support (*p* = 0.002). Specifically, after the first round of clinical training at the first point of measurement, the difference in the quality of mentoring support between students from Group 1 (group mentoring approach) and Group 2 (individual mentoring approach) was not statistically significant (*p* = 0.074). The level of quality of mentoring support increased statistically significantly after the second round of clinical training at the second point of measurement in students from Group 1 who performed clinical training under an individual mentoring approach compared to students from Group 2 who performed clinical training under a group mentoring approach (*p* = 0.017). 

**Figure 2:**
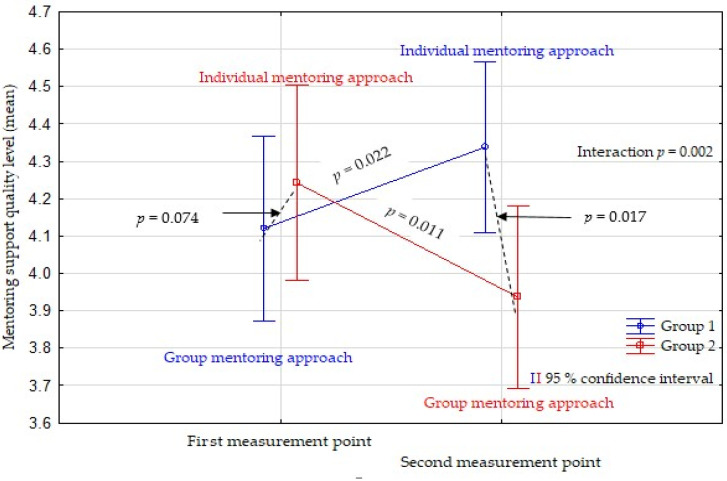
Differences in the quality of mentoring support in the group and individual mentoring approaches.

Furthermore, after the second round of clinical training, at the second measurement point, an increase in the quality of mentoring support was recorded for students from Group 1 (*p* = 0.022) who, after a group mentoring approach in the first round of clinical training, switched to performing clinical training under an individual mentoring approach. Conversely, the quality level of mentoring support dropped significantly (*p* = 0.011) among students from Group 2 after the transfer from the individual mentoring approach in the first round to the group mentoring approach in the second round (Table 1 and Figure 2).

## 4. Discussion

The aim of this study was to investigate the contribution of the mentoring approach during clinical training to the quality level of mentoring support as well as whether the order of application of mentoring approaches (group and individual) contributes to the quality level of mentoring support during clinical training.

The results of this study clearly show that the quality of mentoring support depends not only on the mentoring approach but also on the order of implementation of the individual and group mentoring approaches in clinical training. It is important to emphasise that both after individual and group mentoring in clinical training, and also regardless of the order of application of the group/individual mentoring approaches, an above-average level of quality of mentoring support was recorded. This means that students were generally very satisfied with the mentoring support provided during clinical training, which has also been found in other studies [25,26]. Nursing students are very aware of the importance of clinical training, as it is essential for acquiring the specific competencies required for the nursing profession. This was particularly evident during the recent COVID-19 pandemic when students expressed concern about their professional development because they were unable to undertake clinical training [25,26].

In general, it seems that an individualised mentoring approach has a greater impact on the quality of mentoring support, but this also depends on whether it is implemented as a first or second approach. In particular, students in group 1 who completed their clinical training first in a group and then through an individual mentoring approach expressed a significant increase in the quality level of mentoring support after switching from a group (first round) to an individual mentoring approach (second round). The results were reversed for group 2 students who completed their clinical training first through an individual and then through a group mentoring approach. A significant decrease in the quality level of mentoring support was found after students switched to a group mentoring approach, which is consistent with the results of other studies [2,12,19,27]. Students particularly emphasise the high quality of the relationship between student and mentor in individual mentoring in clinical training [28], the mutual respect with their mentor [2], and the constant feedback from the mentor [12] as the most important factors that make the individual mentoring approach more effective in general. In a recent study conducted in Croatia, nursing students were also most satisfied with the mentoring support provided within the individual mentoring approach and described this approach as the best and most effective compared to the group and dual mentoring approaches [2].

The results of this study are important as they also indicate that the differences in the quality of mentoring support between groups multiply after the second round of clinical training. Indeed, it is evident that the individualised mentoring approach contributes to the increase in the quality of mentoring support, especially after the second round of clinical training. However, this trend of increasing the quality of mentoring support is much more pronounced when the individual approach is followed in the second round, i.e., after the group mentoring approach. In contrast, the quality of mentoring support under the group mentoring approach was significantly lower when the group mentoring approach was applied in the second round after the individual approach. Although the students from Group 1 and Group 2 were trained under different forms of mentoring in the first round of clinical training, the mean values of the quality of mentoring support were more similar after the first round than after the second round of clinical training.

Despite the advantages of an individual mentoring approach during clinical training, it is important to point out that the group mentoring approach also has advantages, such as the possibility of peer support and the adaptation to teamwork [13]. In addition, the results of this study indicate a positive aspect of the group mentoring approach in the initial phase of nursing students’ education, after which the introduction of an individual mentoring approach is definitely recommended, which is a very useful result for effective clinical training planning. However, during clinical training, students certainly experience and recognise the main shortcomings of the group mentoring approach, such as the mentor’s lack of time and the impossibility of adapting his/her approach to the students’ needs [2]. Such situations can potentially lead to a difficult acquisition of knowledge and skills and a feeling of neglect by the mentor [2,11], which certainly affects the evaluation of the quality level of mentoring support. The results of a combined (qualitative and quantitative) study conducted at four Australian universities showed that students were not satisfied with the quality of mentoring support in a group mentoring approach [22], which is confirmed by the results of this study. As reasons for dissatisfaction, students most frequently cited mentor overload and lack of time for students, lack of support from the mentor, and the mentor’s focus on tasks rather than on students’ needs [22]. Similar results were described in the previously aforementioned study conducted in Croatia [2], where nursing students were dissatisfied with the mentor’s support in the group mentoring approach because they did not have the opportunity to think critically and acquire the necessary skills, and because they did not see their mentor as a role model [2], which is one of the extremely important tasks of mentoring [29].

The more prominent difference in the quality of mentoring support between individual and group mentoring approaches after the second round of clinical training could be related to students recognising the true differences, advantages, and disadvantages of the group and individual mentoring approaches after completing both rounds of clinical training and undertaking clinical exercises with both group and individual mentoring approaches. Therefore, students were able to experience, analyse, and critically evaluate both mentoring approaches during clinical training. It is therefore possible that the students in Group 1, who initially completed clinical training with a group mentoring approach, were able to experience and learn about the specific benefits of an individual mentoring approach in the second round of clinical training, which led to a significant increase in their assessment of the quality of mentoring support. In contrast, the students in Group 2, who practiced an individual mentoring approach in the first round of clinical training and gave it an above-average rating, only recognised and appreciated the actual benefits of a group mentoring approach after completing the clinical training, which led to a significant decrease in their rating of the quality of mentoring support and a significantly lower rating of the quality of support in the group mentoring approach. The results confirm the benefits of an individual mentoring approach during clinical training in terms of students’ perception and evaluation of the quality of mentoring support, but only after students have had both group and individual mentoring experiences. Finally, we can conclude that the results of this study suggest that, in addition to the form of the mentoring approach, it is also important to plan the sequence of changes in the mentoring approach when planning and implementing clinical training, whereby the group mentoring approach should precede individual mentoring.

The results of this study confirm previous findings and statements about the importance of the mentoring approach during clinical training and emphasise the individual mentoring approach as more dominant when it comes to achieving a higher quality of mentoring support [30,31,32,33,34]. However, the results suggest that a group mentoring approach should be used at the beginning of clinical training. Thereafter, it is recommended to switch to an individual mentoring approach, as this has a stronger influence on the experience of the quality of mentoring support. These findings can provide valuable guidance for mentors in designing and coordinating clinical education.

Despite the value of this quasi-experimental study, one of the limitations is the small sample size at only one university in Croatia. The allocation of students to Group 1 and Group 2 was not randomised; they belonged to the respective academic year. Nevertheless, a complete equalisation of the groups was not possible, but the students were divided into groups based on grades and study satisfaction, which are assumed in the literature to influence the dependent variable. The period between the first and second mentoring approaches refers to the fact that the time interval during which different methods of mentoring were applied was relatively short, limited by the duration of the academic year, and it is possible that not all the effects of the previously applied mentoring approach were removed. During the study, it was not possible to fully control for other conditions that might influence the dependent variable, such as the characteristics of the individual mentors and/or the type of clinical settings that prevailed during clinical training in the workplace. Finally, it is recommended for future research to include a larger number of students from multiple universities as well as a longer duration of clinical training, which could further strengthen the research results.

## 5. Conclusions

The results of this study showed that students exposed to different mentoring approaches reported different quality levels of mentoring support. Students made the best estimate of the individual mentoring approach, especially if it was performed after the group mentoring approach. Respecting the previously known advantages and disadvantages of both applied mentoring approaches, the recorded results can facilitate the decision of educational institutions on the combination of both mentoring approaches. Also, the results contribute to a better understanding of the needs for individual or the combined use of group and individual mentoring approaches to achieve the desired outcomes as successfully as possible. The aforementioned can significantly facilitate the process of planning, implementation, evaluation, and ultimately adaptation of the performance of clinical training to the individual needs of students and increase the quality of mentoring support.

## Figures and Tables

**Table 1:** Quality levels of mentoring support in the group and individual mentoring approaches.

Group	n	First Round		n	Second Round	
		Mentoring Approach	Mean (SD)		Mentoring Approach	Mean (SD)
Group 1	60	GMA	4.12 (0.94)	53	IMA	4.34 (0.91)
Group 2	59	IMA	4.24 (0.66)	54	GMA	3.94 (1.04)

SD—standard deviation; GMA—group mentoring approach; IMA—individual mentoring approach.

## Data Availability

Data supporting the published results can be obtained from the corresponding authors.

## Public Involvement Statement

There was no public involvement in any aspect of this research.

## Guidelines and Standards Statement

This manuscript was drafted against the STROBE Statement for reports of cohort studies.
